# Monocarboxylate transporter 4 involves in energy metabolism and drug sensitivity in hypoxia

**DOI:** 10.1038/s41598-023-28558-4

**Published:** 2023-01-27

**Authors:** Atsushi Yamaguchi, Yuto Mukai, Tomoya Sakuma, Katsuya Narumi, Ayako Furugen, Yuma Yamada, Masaki Kobayashi

**Affiliations:** 1 Department of Pharmacy, Hokkaido University Hospital, Kita-14-Jo, Nishi-5-Chome, Kita-ku, Sapporo, 060-8648 Japan; 2 Laboratory of Clinical Pharmaceutics and Therapeutics, Division of Pharmasciences, Faculty of Pharmaceutical Sciences, Hokkaido University, Kita-12-Jo, Nishi-6-Chome, Kita-ku, Sapporo, 060-0812 Japan; 3 Education Research Center for Clinical Pharmacy, Faculty of Pharmaceutical Sciences, Hokkaido University, Kita-12-Jo, Nishi-6-Chome, Kita-ku, Sapporo, 060-0812 Japan; 4 Laboratory for Molecular Design for Pharmaceutics, Faculty of Pharmaceutical Sciences, Hokkaido University, Kita-12-Jo, Nishi-6-Chome, Kita-ku, Sapporo, 060-0812 Japan

**Keywords:** Carrier proteins, Cancer metabolism, Cancer microenvironment, Cancer therapy

## Abstract

Metabolic reprogramming of cancer cells is a potential target for cancer therapy. It is also known that a hypoxic environment, one of the tumor microenvironments, can alter the energy metabolism from oxidative phosphorylation to glycolysis. However, the relationship between hypoxia and drug sensitivity, which targets energy metabolism, is not well known. In this study, A549 cells, a cell line derived from lung adenocarcinoma, were evaluated under normoxia and hypoxia for the sensitivity of reagents targeting oxidative phosphorylation (metformin) and glycolysis (α-cyano-4-hydroxycinnamic acid [CHC]). The results showed that a hypoxic environment increased the expression levels of monocarboxylate transporter (MCT) 4 and hypoxia-induced factor-1α (HIF-1α), whereas MCT1 and MCT2 expression did not vary between normoxia and hypoxia. Furthermore, the evaluation of the ATP production ratio indicated that glycolysis was enhanced under hypoxic conditions. It was then found that the sensitivity to metformin decreased while that to CHC increased under hypoxia. To elucidate this mechanism, MCT4 and HIF-1α were knocked down and the expression level of MCT4 was significantly decreased under both conditions. In contrast, the expression of HIF-1α was decreased by HIF-1α knockdown and increased by MCT4 knockdown. In addition, changes in metformin and CHC sensitivity under hypoxia were eliminated by the knockdown of MCT4 and HIF-1α, suggesting that MCT4 is involved in the phenomenon described above. In conclusion, it was shown that the sensitivity of reagents targeting energy metabolism is dependent on their microenvironment. As MCT4 is involved in some of these mechanisms, we hypothesized that MCT4 could be an important target molecule for cancer therapy.

## Introduction

Metabolic reprogramming is recognized as one of the hallmarks of cancer and is a target for cancer therapy^1^. Although aerobic glycolysis, known as the Warburg effect, is considered the characteristic metabolic state of cancer cells^2^, the reverse Warburg effect, in which oxidative cancer cells use lactate produced by glycolysis of surrounding fibroblasts as fuel for oxidative phosphorylation (OXPHOS), has recently been proposed as another metabolic characteristic of cancer cells^3^. Similarly, the metabolic response from OXPHOS to glycolysis in hypoxic environments in cancer cells is mediated by the stabilization of hypoxia-induced factor-1α (HIF-1α), which is an important factor in cancer metabolism and therapeutic targets^4^. However, the effects of hypoxic environment on drug sensitivity to energy metabolism remain unclear.

Metformin, an antidiabetic drug, has been shown to have anticancer effects^5^. The detailed mechanism of its anticancer activity is not clear, but it has various actions, one of which is the suppressive effects on OXPHOS mediated by the inhibition of mitochondrial respiratory chain complex I^6^. In a previous study, though metformin showed potent antitumor effects on those cancer cells that obtain energy from OXPHOS, its inhibitory effects were reduced in cells related to aerobic glycolysis^7^. This suggests that the sensitivity of oxidative-glycolysis cancer cells to these inhibitors is dependent on the metabolic performance of cancer cells.

Lactate, a metabolite of glycolysis and a source of energy for oxidative cancer cells, is a significant molecule, and monocarboxylate transporters (MCTs) transfer lactate across the plasma membrane into and out of the cell^8^. MCTs belong to the SLC16 family and consist of 14 isoforms^9^. Among them, MCT1, MCT2, and MCT4 are expressed in cancer cells, and high MCT4 expression indicates poor survival in various cancer types^10^. MCT1 and MCT2 are thought to contribute to lactate uptake by oxidative cancer cells, whereas MCT4 is thought to contribute to the extracellular release of lactate produced by glycolysis^11^. In addition, MCT4 is highly expressed in glycolytic cancer cells and supports their proliferation^12^. Moreover, MCTs are involved in Warburg and reverse Warburg effects^3,13^ and thus, aid in adaptation to hypoxic environment^14^. Consequently, MCT inhibitors are candidate anticancer drugs and several compounds in this regard have been reported^15,16^. α-Cyano-4-hydroxycinnamic acid (CHC), a classic and nonselective inhibitor of MCTs, has been shown to have antineoplastic effects and may accumulate lactate in cancer cells and suppress glycolysis^17,18^.

Considering this background, this study hypothesized that metabolic reprogramming from the OXPHOS preference phenotype to the glycolysis-dominant phenotype induced by a hypoxic environment would alter the antineoplastic effects of OXPHOS/glycolysis-targeting drugs. To test this hypothesis, A549 cells, a cell line derived from lung adenocarcinoma, were used because they show preference for OXPHOS metabolism compared with other lung adenocarcinoma cell lines^7^. Cells were cultured under normoxia or hypoxia, and their sensitivity to metformin as an OXPHOS inhibitor and CHC as a glycolysis inhibitor was evaluated.

## Results

### Hypoxia upregulated MCT4 and HIF-1α expression

**Figure 1:**
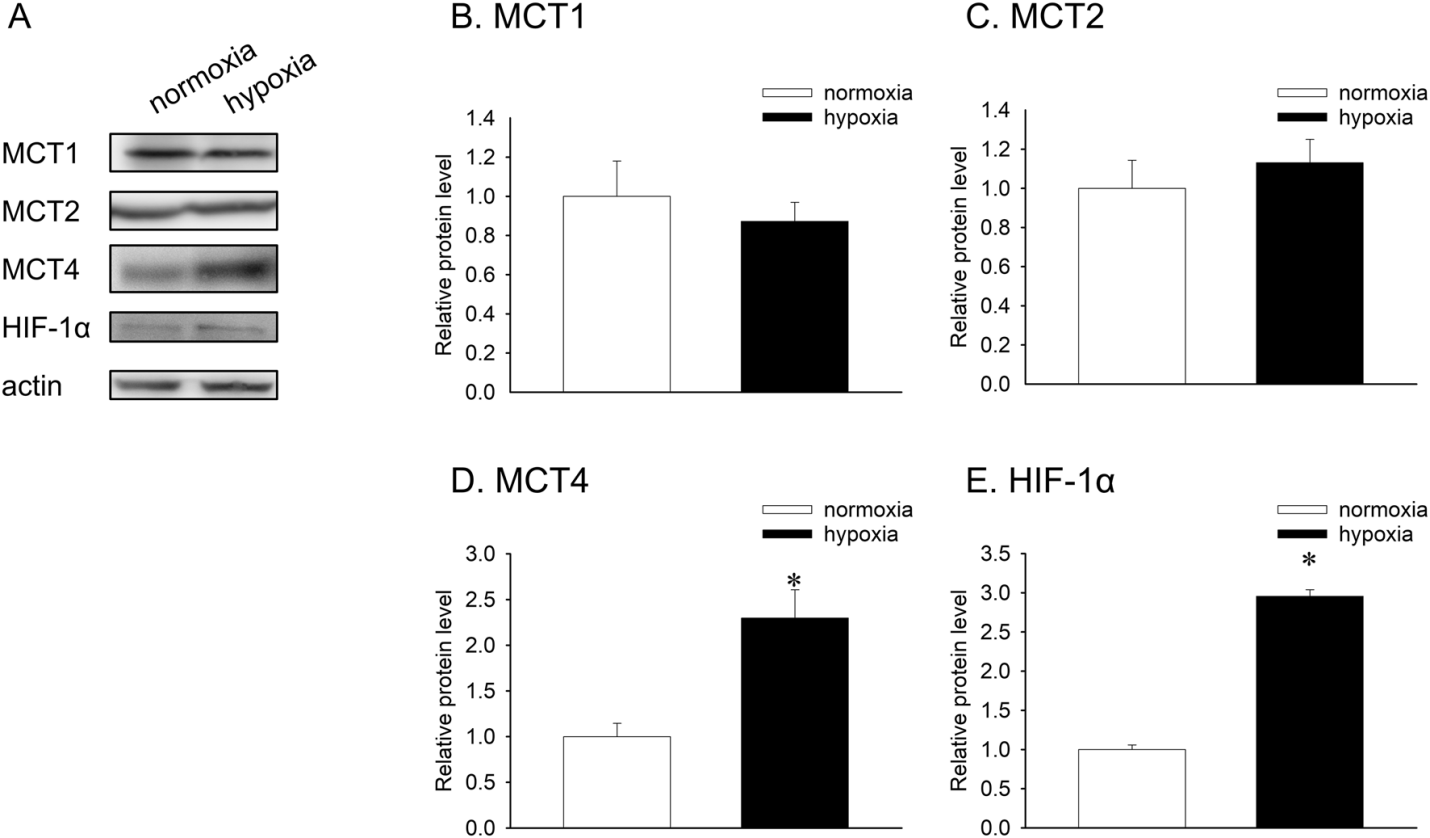
Protein expression level of MCT1, MCT2, MCT4, and HIF-1α in normoxia and hypoxia. (**A**) Western blot analysis of MCT1, MCT2, MCT4, and HIF-1α in normoxia and hypoxia in A549 cells. The protein expression levels of MCT1 (**B**), MCT2 (**C**), MCT4 (**D**), and HIF-1α (**E**) were visualized, respectively. Data are presented as mean ± standard error of three independent experiments. *p < 0.05 compared with the normoxia using unpaired Student’s* t* test.

**Figure 2:**
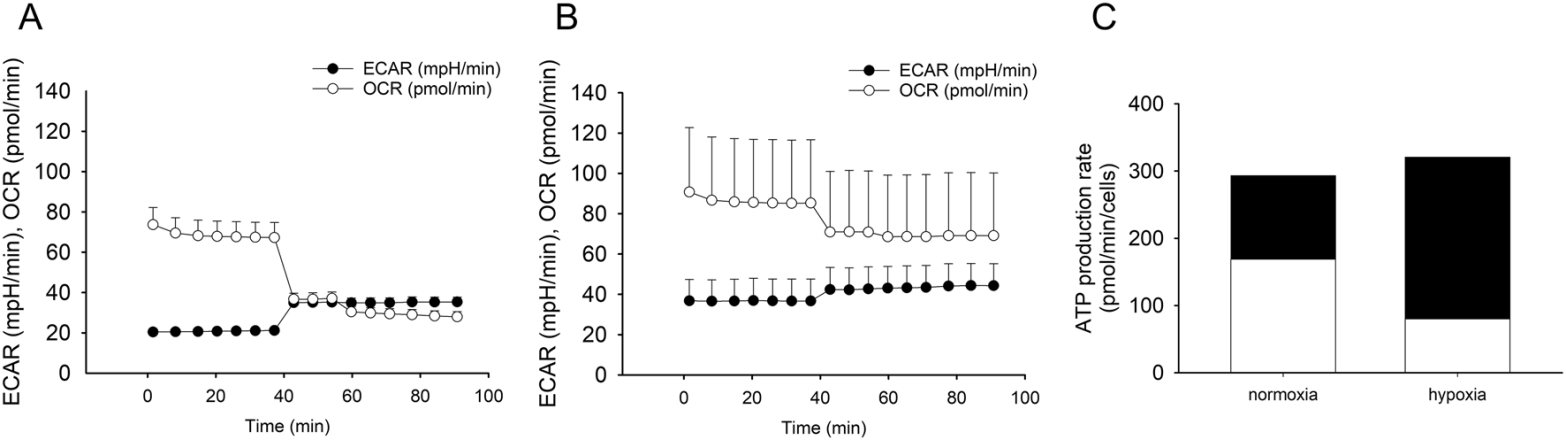
ATP production rate in normoxia and hypoxia. Mitochondrial respiratory activity was evaluated using a Seahorse XFp analyzer. The oxygen consumption rate (OCR) and extracellular acidification rate (ECAR) were calculated by normalizing the cell counts under normoxia (**A**) and hypoxia (**B**). Each mark represents the mean with a positive SE of three or four independent experiments. (**C**) ATP production rate under normoxia and hypoxia calculated from (**A**,**B**), respectively. Each mark represents the mean of three or four independent experiments.

To confirm hypoxia-induced metabolic alterations, A549 cells were cultured under normoxia and hypoxia and the protein expression levels of MCT1, MCT2, MCT4, and HIF-1α (Fig. 1A, Supplementary Fig. 1) were evaluated. A significant increase in MCT4 and HIF-1α expression under hypoxia was observed compared to normoxia, while MCT1 and MCT2 expression did not differ between normoxia and hypoxia (Fig. 1B–E). In addition, an XF analyzer was used to measure the rate of glycolysis and oxidative ATP production (Fig. 2). Under normoxic conditions, A549 cells obtained ATP primarily via oxidative metabolism. In contrast, hypoxia changed the phenotype of A549 cells from oxidative to glycolytic (Table 1). Therefore, it was hypothesized that hypoxia induces metabolic changes from OXPHOS to glycolysis.

**Table 1:** ATP production rate in normoxia and hypoxia.

	% of total ATP production rate		*p* value by *t* test
	Normoxia (mean ± S.E.)	Hypoxia (mean ± S.E.)	
OCR (oxidative)	49.7 ± 4.5	22.6 ± 6.2	< 0.05
ECAR (glycolysis)	50.3 ± 4.5	77.4 ± 6.2	< 0.05

Mitochondrial respiratory activity was evaluated using a Seahorse XFp analyzer. The oxygen consumption rate (OCR) and extracellular acidification rate (ECAR) represent oxidative and glycolysis phenotypes, respectively. Statistical analyses were performed using unpaired Student’s *t* test.

### Hypoxia attenuated the effects of OXPHOS-targeting inhibitors and enhanced the effects of glycolysis-targeting inhibitors

**Figure 3:**
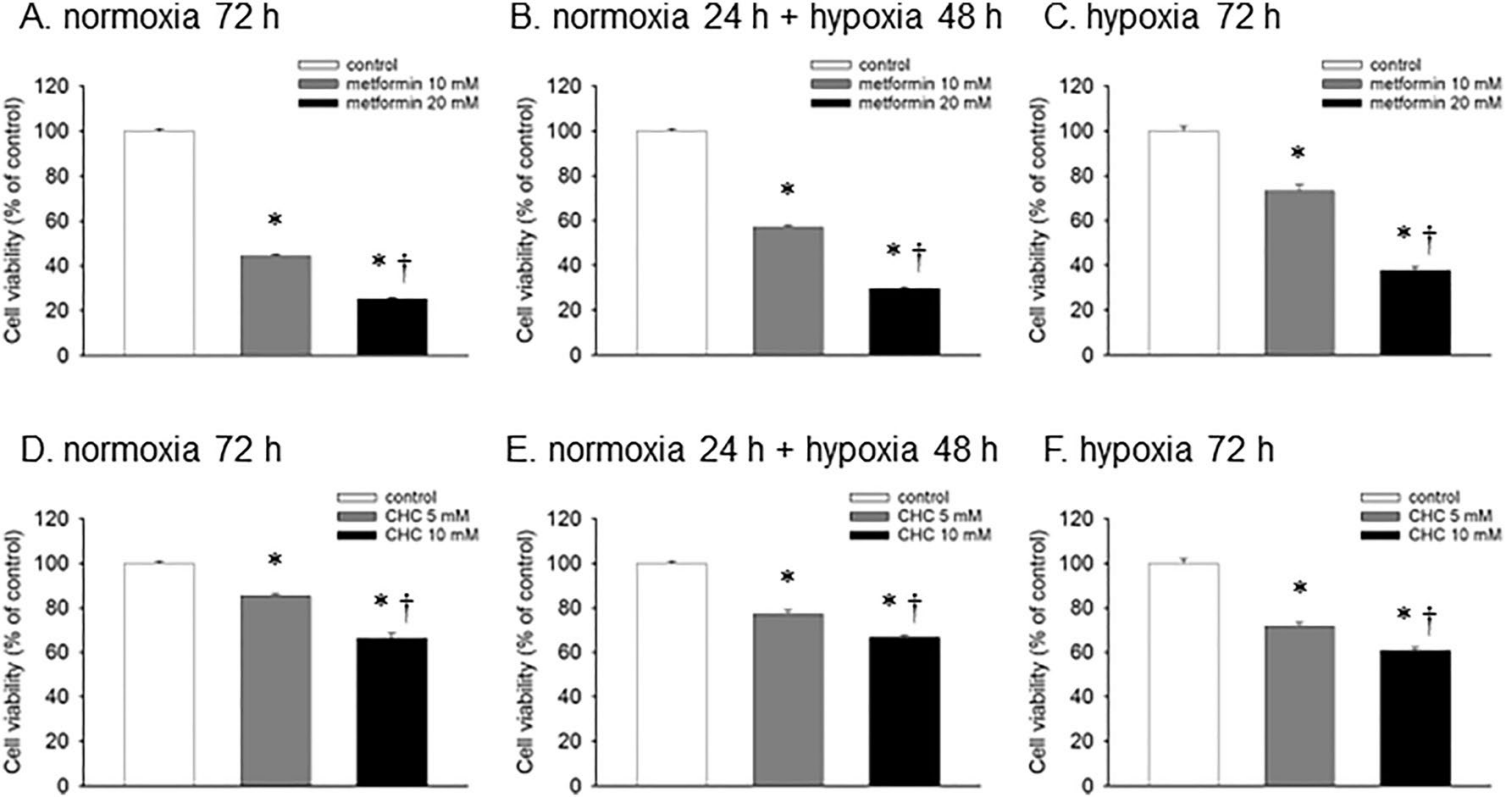
Effects of metformin and CHC on cell viability with concentration dependency of each reagent and time dependency of hypoxic dependency. Concentration dependence of metformin 10 mM and 20 mM (**A**–**C**) and CHC 5 mM and 10 mM (**D**–**F**) for cell viability using the MTT assay. Time dependence of cell viability under hypoxic conditions (normoxia 72 h, normoxia 24 h + hypoxia 48 h, and hypoxia 72 h) using the MTT assay. Data are presented as the mean ± standard error of three independent experiments. *p < 0.05, compared with the control; ^†^*p* < 0.05, compared with metformin 10 mM or CHC 5 mM using the Tukey–Kramer test.

An MTT assay was used to determine the antitumor effects of the OXPHOS-targeting inhibitor metformin and glycolysis-targeting inhibitor CHC under various oxygen conditions (Fig. 3). The cell viability was decreased by metformin and CHC in a concentration-dependent manner under all oxygen conditions. Notably, the degree of anticancer effects induced by 10 mM metformin was reduced under hypoxia in a time-dependent manner (44.6%, 57.2%, and 73.2% under normoxia for 72 h, normoxia for 24 h + hypoxia for 48 h, and hypoxia for 72 h, respectively) (Fig. 3A–C). In contrast, the opposite results were observed with the addition of 5 mM CHC (85.4%, 77.3%, and 71.7% under normoxia for 72 h, normoxia for 24 h + hypoxia for 48 h, and hypoxia for 72 h, respectively) (Fig. 3D–F), which is consistent with the hypothesis that drug sensitivity varies in response to altered metabolic conditions in a hypoxic environment.

### MCT4 expression was involved in the alteration of drug sensitivity in hypoxic conditions

**Figure 4:**
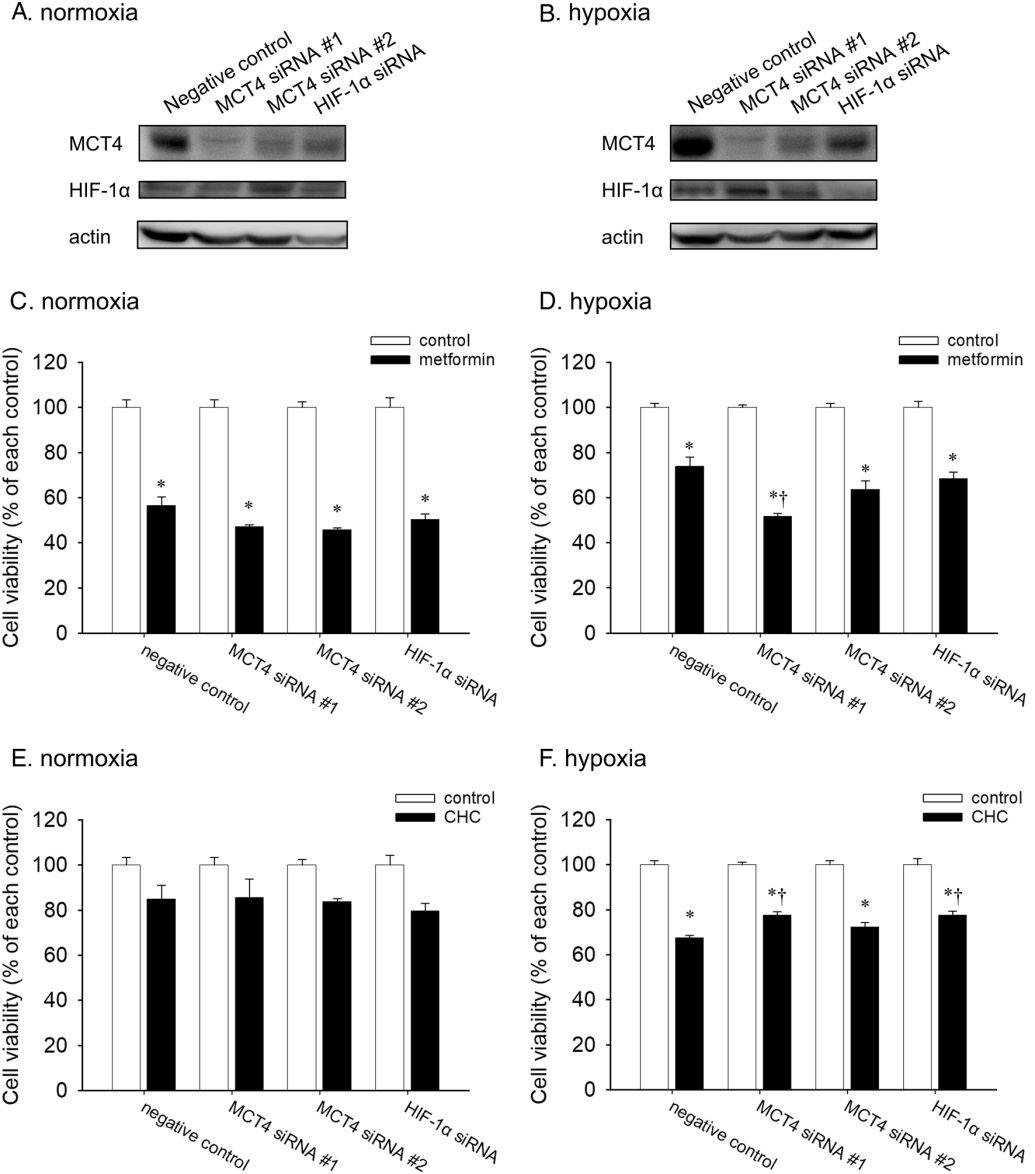
Effects of MCT4 and HIF-1α siRNA on protein expression level and sensitivity of metformin and CHC. Western blot analysis after transfection with MCT4 and HIF-1α siRNA under normoxia (**A**) and hypoxia (**B**). Cell viability after treatment with 10 mM metformin under normoxia (**C**) and hypoxia (**D**) in A549 cells transfected with siRNA. Viability of CHC 5 mM under normoxia (**E**) and hypoxia (**F**) in A549 cells transfected with siRNA. Data are presented as the mean ± standard error of three independent experiments. *p < 0.05, compared with the control; ^†^*p* < 0.05, compared with metformin 10 mM or CHC 5 mM using the Tukey–Kramer test.

Hypoxia increased the expression of MCT4 and HIF-1α and altered energy metabolism, which may induce changes in the sensitivity of metformin and CHC on A549 cells. Therefore, the reagent sensitivity of A549 cells transfected with MCT4 and HIF-1α siRNAs was examined. Western blotting analysis showed that the expression of HIF-1α was decreased by HIF-1α siRNA and increased by MCT4 siRNA, particularly under hypoxia (Fig. 4A,B, Supplementary Fig. 2). In contrast, MCT4 expression was decreased by all the siRNAs, with MCT4 siRNA#1 being the most potent (Supplementary Fig. 3). Consequently, HIF-1α positively regulates MCT4 expression.

Finally, the effects of metformin and CHC on the viability of the siRNA-transfected A549 cells were evaluated. In normoxia, knockdown of MCT4 and HIF-1α had no impact on the sensitivity to metformin or CHC (Fig. 4C,E). Interestingly, the sensitivity of A549 cells to metformin under hypoxic conditions increased significantly with MCT4 siRNA #1 treatment (Fig. 4D). Moreover, CHC sensitivity significantly decreased in A549 cells transfected with MCT4 siRNA#1 and HIF-1α siRNA (Fig. 4F). Therefore, MCT4 expression may be associated with hypoxia-induced alterations in drug sensitivity.

## Discussion

Metabolic reprogramming including MCTs that are involved in lactate shuttling between glycolytic and oxidative cancer cells, is a new target for cancer therapy. The microenvironment (for e.g., hypoxia) of tumor cells, is also an important factor in the energetic metabolism of cancer cells. This experiment demonstrated the hypoxia-induced enhancement of MCT4 and HIF-1α protein expression (Fig. 1A,D,E). In addition, knockdown experiments showed that HIF-1α positively regulates MCT4 expression. These results are in agreement with those of the previous studies^4,19^. In addition, the ATP production ratio of A549 cells was converted from OXPHOS to glycolysis under hypoxia (Fig. 2), which indicated that hypoxia directly induced metabolic alterations. Furthermore, sensitivity to metformin was attenuated and sensitivity to CHC was enhanced by hypoxia. These results are also coordinated with the inhibition targets of these reagents. In terms of the effects of siRNA, a decrease in MCT4 expression did not change reagent sensitivity under normoxic conditions, which indicated that MCT4 does not function to release extracellular lactate in normoxia because pyruvate and lactate are poorly produced by glycolysis and are consumed by OXPHOS. In contrast, under hypoxia, MCT4 contributes to the release of lactate accumulated by glycolysis and regulates the alteration of energy metabolism. Therefore, MCT4 could counteract the variability in metformin and CHC sensitivity in hypoxia, and may play an important role in the response to hypoxia and the alteration of energy metabolism. Based on these results, we concluded that MCT4 could be a therapeutic target for cancer via energy metabolism.

Metformin inhibits OXPHOS and exerts antineoplastic effects. In the present study, the efficacy of metformin was attenuated under hypoxic conditions (Fig. 3A–C). Previous studies on metformin sensitivity in various lung cancer cell lines have shown that this effect is more pronounced in cell lines with predominant OXPHOS^7^. Similarly, in colorectal cancer, the sensitivity to metformin varies depending on the metabolic state of the cells, and it has been confirmed that metformin has a weak effect on phenotypes in which the glycolytic system is dominant^20^. In addition, low glucose levels increased the sensitivity to metformin, and this phenomenon was more pronounced in cells with predominant OXPHOS^21^. These phenomena seem reasonable because energy production under OXPHOS at low glucose levels is more dominant than that in the glycolytic system. In contrast, the present study evaluated the changes from OXPHOS to glycolysis induced by hypoxia. Other studies on metformin sensitivity have reported that mitochondrial function was enhanced in cisplatin-resistant cell lines, and metformin-induced inhibition of oxidative phosphorylation was improved in these resistant cells^22^. Two other enzymes important for cancer metabolism are lactate dehydrogenase (LDH) A and LDH B, which catalyze the formation of pyruvate to lactate, and the reverse reaction, respectively^23^. LDHA/B double knockout causes a shift to OXPHOS, low proliferation in hypoxia, and sensitization to phenformin, another mitochondrial complex I inhibitor^24^. Thus, the inhibition of OXPHOS by metformin is more effective in cells with a predominance of OXPHOS, suggesting that the intensity of the effect of metformin varies depending on the metabolic state of cancer cells. Metformin has been reported to improve progression-free survival in meta-analyses of advanced lung cancer^25^. In contrast, a randomized controlled trial indicated that metformin lowered the survival outcomes of patients with locally advanced NSCLC^26^. These clinical results also suggest that the efficacy of metformin depends on the cancer type and staging.

Since MCT1, MCT2, and MCT4 are highly expressed in cancer cells, inhibitors of these are potential anticancer drugs. AZD3965, a dual inhibitor of MCT1 and MCT2, is at phase I trial for advanced cancer. In a previous study using small cell lung cancer cell lines, it was reported that sensitivity to AZD3965 varied between cell lines^27^. In addition, cell lines that did not express MCT4 showed increasing sensitivity to MCT1 inhibition by AZD3965 under hypoxia; whereas cell lines that expressed MCT4, whose expression increased under hypoxia showed resistance to AZD3965^27^. In addition, MCT4 expression abolished glycolysis suppression induced by AR-C155858, another MCT1/2 dual inhibitor, and attenuated the antitumor effects of AR-C155858^28^. Taken together with the results of this study, it is possible that MCT1 is involved in the response of energy metabolism to hypoxia in cells that do not express MCT4, and that cells are therefore more sensitive to its inhibitors. However, in cells that express MCT4, MCT4 responds to hypoxia and the effects of MCT1 inhibition are no longer observed. Furthermore, Noble et al. (2017) reported that the combination of AZD3965 and metformin caused significant cell death in a Hodgkin’s lymphoma cell line in which MCT1 was predominantly expressed^29^, which is appropriate because the energy derived from glycolysis and OXPHOS was significantly inhibited. A similar phenomenon has been observed in diffuse large B-cell lymphoma that lacks MCT4 expression, and the simultaneous targeting of glycolysis and oxidative phosphorylation by the combination of AZD3965 and IACS-010759, an inhibitor of mitochondrial complex I, was recently reported to be more effective^30^. Furthermore, Epstein-Barr virus (EBV) infection promotes B lymphoid tumors, and EBV-infected B cells upregulate MCT4, resulting in resistance to AZD3965^31^. In this case, VB124, an MCT4 inhibitor, had antitumor effect, and the combination of AZD3965 and VB124 sensitized the killing by metformin^31^. Consequently, the simultaneous inhibition of MCTs and mitochondrial respiration has strong fatal effects against tumor cells^32^. This study revealed that the classical MCTs non-selective inhibitor CHC suppressed the viability of A549 cells. The sensitivity of CHC increased under hypoxia with the upregulation of MCT4, which indicated that MCT4 was involved in the alteration of energy metabolism induced by hypoxia.

In conclusion, we demonstrated that hypoxia induced metabolic alterations from OXPHOS to glycolysis and altered the sensitivity of metabolic targeting inhibitors. MCT4 is partially involved in the mechanisms underlying these phenomena. To the best of our knowledge, this is the first study to reveal the association between oxygen conditions and drug sensitivity in inhibiting energy metabolism. These results will contribute to cancer therapies that target MCT4. However, this study has a major limitation as for reproducibility because we performed experiment only on A549 cell line. In this paper, we used normoxic and hypoxic A549 cell line to compare the drug sensitivity in oxygen states. Similarly, A549 cell line was used to evaluate glycolysis and OXPHOS under various conditions in recent articles^33,34^. In addition, the energy metabolic adaptation to environmental condition were similar in multiple cancer cells including A549 cells^35^. Furthermore, A549 cell line was one of the most common used lung cancer cells and was evaluated for the regulation of MCTs expression^36,37^. Therefore, we believe that our report is valuable. Although the metabolic characteristics of A549 cell line are identified among other lung cancer cell lines^7^, further research using other cancer cell lines is required to substantiate our findings.

## Materials and methods

### Chemicals

Metformin was purchased from Enzo Biochem (New York, NY, USA) and CHC was purchased from Sigma-Aldrich (St. Louis, MO, USA). All the compounds used were of the highest purity available.

### Cell culture

A549 cells (American Type Culture Collection, Manassas, VA, USA) were grown in Dulbecco’s Modified Eagle’s medium (DMEM) supplemented with 10% fetal bovine serum (FBS) and 1% penicillin–streptomycin (Sigma-Aldrich, St. Louis, MO, USA) at 37 °C in a 5% CO_2_ atmosphere under normoxia and hypoxia (5% O_2_). A BIONEZ-3 hypoxia culture kit (Sugiyamagen, Tokyo, Japan) was used to determine the hypoxic environment.

### Western blotting

Cells were lysed in a lysis buffer supplemented with 60 mM Tris–HCl (pH 6.8) and 1% sodium dodecyl sulfate (SDS) in distilled water. The lysate was stored on ice for 5 min and sonicated briefly at 4 °C. The sample was centrifuged at 12,000×*g* for 15 min at 4 °C and the clear supernatant was collected. The protein concentration was determined using a Pierce® bicinchoninic acid (BCA) protein assay kit (Thermo Fisher Scientific). All lysates were added to loading buffer containing 0.1 M tris-hydrochloride, 4% SDS, 10% 2-mercaptoethanol, 20% glycerol, and 0.004% bromophenol blue. Proteins (40 μg) were subjected to sodium dodecyl sulfate–polyacrylamide gel electrophoresis (SDS-PAGE) and electrophoretically transferred onto polyvinylidene difluoride (PVDF) membranes (Bio-Rad). The membranes were blocked with PBS containing 0.05% Tween 20 (PBS/T) and 1% non-fat dry milk for 1 h at room temperature. After washing with PBS/T, the membranes were incubated overnight at 4 °C with primary antibodies against MCT1 (1:200, SC-365501, Santa Cruz Biotechnology), MCT2 (1:1000, ab198272, Abcam), MCT4 (1:1000, 22787-1-AP, Proteintech), HIF-1α (1:200, sc-10790, Santa Cruz Biotechnology), and actin (1:1000, ab179467, Abcam). The membranes were cut prior to hybridization with antibodies in HIF-1α and actin. The bands were detected using horseradish peroxidase (HRP)-conjugated secondary antibodies (1:4000) and visualized using ECL Western Blotting Detection Reagents (Cytiva).

### Measure of ECAR and OCR

The extracellular flux analyzer provided a continuous quantitative assessment of mitochondrial respiration, as previously reported^38–40^. Seahorse XFp (Agilent Technologies, Santa Clara, CA, USA) was used to measure the mitochondrial oxygen consumption rate (OCR) and extracellular acidification rate (ECAR) of A549 cells using a Seahorse XF Real-Time ATP Rate Assay Kit. These parameters were estimated by the sequential addition of oligomycin (an inhibitor of ATP synthesis), rotenone, and antimycin A (complete inhibition of mitochondrial respiration), and the OCRs and ECARs were measured.

At 48 h before the start of the experiment, 10,000 cells per well were seeded into 8-well plates and incubated at 37 °C under an atmosphere of 5% CO_2_ under normoxia and hypoxia (5% O_2_). Prior to analysis, the cells were incubated in analytical medium at 37 °C and CO_2_-free for 1 h. After measuring the baseline OCRs (pmol/min) and ECARs (mpH/min), oligomycin (final concentration of 2 μM), rotenone (final concentration, 0.5 μM), and antimycin A (final concentration of 0.5 μM) were continuously added to the cells. The obtained data (Fig. 2A,B) were used to determine the ATP production rate (pmol/min/cell) (Fig. 2C, Table 1).

### MTT assay

Cell viability was evaluated using the 3-(4,5-dimethylthiazol-2-yl)-2,5-diphenyltetrazolium bromide (MTT) assay. 24 h after seeding, various concentrations of metformin and CHC were added and incubated for 48 h. The MTT solution was added to the medium, and the cells were further incubated for 1 h. The MTT medium was then replaced with dimethyl sulfoxide (DMSO), and the absorbance was measured at 590 nm.

### RNA interference

The siRNA transfection was optimized using Lipofectamine RNAiMAX (Invitrogen). Silencer Select siRNA against MCT4 (#1; s17416 and #2; 117233), HIF-1α (42840), and negative control siRNA (Silencer Negative Control No. 1 siRNA) were purchased from Thermo Fisher Scientific. A549 cells were transfected with siRNA via reverse transfection at a final concentration of 10 nM. After siRNA transfection (24 h), cells were used for various experiments after 48 h.

### Statistical analysis

Statistical analyses of the data were performed using the unpaired Student’s *t* test or Tukey–Kramer test. Data were analyzed using SigmaPlot 14.5, and differences were considered statistically significant at *p* < 0.05.

## Supplementary Information

Supplementary Figure 1.

Supplementary Figure 2.

Supplementary Figure 3.

Supplementary Legends.

## Data Availability

The datasets generated and/or analyzed during the current study are available from the corresponding author upon reasonable request.
