# Supplementary material for: Monocarboxylate transporter 4 involves in energy metabolism and drug sensitivity in hypoxia
Source: Sci Rep. 2023 Jan 27;13:1501. doi: 10.1038/s41598-023-28558-4 (PMC9883486; doi:10.1038/s41598-023-28558-4)
Supplement: Supplementary file 4 — Supplementary Legends. [file 41598_2023_28558_MOESM4_ESM.docx]

Fig. S1. Full length of western blot analysis of MCT1 (A), MCT2 (B), MCT4 (C), and HIF-1α (D) and actin (E). We described some replicates of western bolt in full length (left) and membrane adjusting brightness/contract in entire image to be visible for membrane edges (right). The membranes were cut prior to hybridization with antibodies in HIF-1α and actin. The lanes shown in red frames were used in Figure 1.

Fig. S2. Full length of western blot analysis evaluating siRNA knockdown of MCT4 and HIF-1α. We described western bolt in full length (left) and membrane adjusting brightness/contract in entire image to be visible for membrane edges (right). Western blot of MCT4 (A), HIF-1α (B), and actin (C) under normoxic and hypoxic conditions. The membranes were cut prior to hybridization with antibodies in HIF-1α and actin. The lanes shown in red frames were used in Figure 4.

Fig. S3. Effect of siRNA knockdown of MCT4 and HIF-1α. Protein expression levels of MCT4 (A, C) and HIF-1α (B, D) after siRNA transfection under normoxic and hypoxic conditions. The data are taken from Figure 4A and B.
